# 3D Proteomics: Structural, Functional, Chemical and Biomarker Discovery Proteomics With LiP-MS

**DOI:** 10.1016/j.mcpro.2026.101552

**Published:** 2026-03-06

**Authors:** Franziska Elsässer, Natalie de Souza, Paola Picotti

**Affiliations:** Institute of Molecular Systems Biology, ETH Zurich, Zurich, Switzerland

**Keywords:** chemical proteomics, functional proteomics, limited proteolysis, mass spectrometry, structural proteomics, structural systems biology

## Abstract

Protein structural dynamics drive changes in protein function, making the capture of such dynamics essential for interrogating biological systems. Here we review limited proteolysis coupled to mass spectrometry (LiP-MS), a structural and chemical proteomics method that uses changes in susceptibility to protease cleavage to profile proteome-wide protein structural changes within complex biological samples. In the decade since its development, LiP-MS has become a broadly used structural proteomics method, with peptide-level resolution. It has identified drug targets, delineated altered cellular pathways in response to complex perturbations, revealed structural information on otherwise challenging protein targets, and demonstrated the new concept of structural biomarkers of disease. Because LiP-MS simultaneously probes numerous types of molecular events, such as molecular binding, changes in enzyme activity, chemical modifications, allosteric conformational changes, aggregation, and unfolding, it supports a new proteomics workflow which we term 3D proteomics. This workflow enables the detection of specific functional sites within proteins that are altered upon perturbation, thereby guiding the generation of molecular hypotheses. Further, by globally profiling structural in addition to protein abundance changes, LiP-MS has proven able to greatly increase the information content of functional proteomics screens. In sum, LiP-MS has supported the development of a novel conceptual framework for generating, visualizing, and interpreting structural proteomics data with peptide level resolution, thereby comprehensively probing biological systems. Here we survey the applications of LiP-MS, discuss methodological variants developed by us and others, and describe the use of this new type of omics readout for structural, functional, chemical, and biomarker discovery proteomics.

## Highlights

• LiP-MS achieves global profiling of protein structural changes in complex samples.
• Detects molecular events with peptide-level resolution.
• Identifies drug targets, disease biomarkers, and altered pathways upon perturbation.
• Improves the information content of functional proteomics screens.
• Enables 3D proteomics, a novel framework for interpreting structural proteomics data.

## Graphical Abstract

**Figure:**
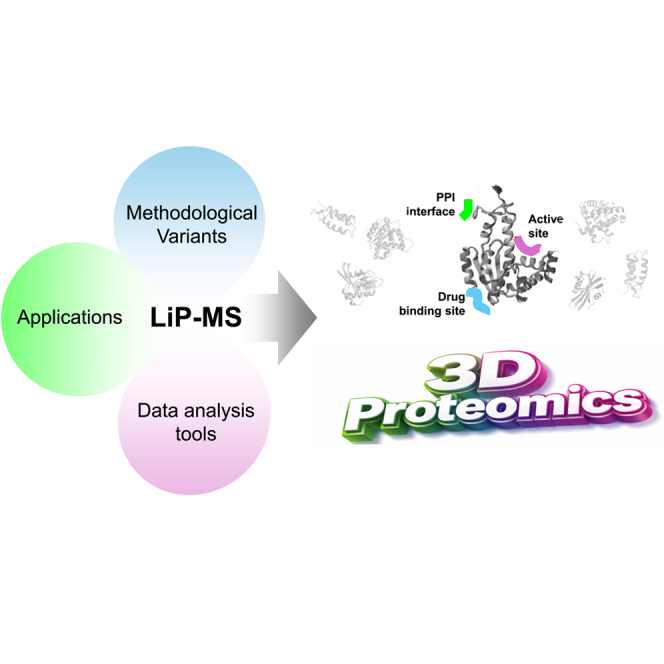

## In Brief

We review limited proteolysis coupled to mass spectrometry (LiP-MS), a structural and chemical proteomics method to profile proteome-wide protein structural changes within complex biological samples. We provide an overview of methodological variants of LiP-MS, including their pros and cons, and survey its applications to biological questions over the past decade. We illustrate how LiP-MS enables 3D proteomics, a novel type of omics readout for structural, functional, chemical, and biomarker discovery proteomics.

The goal of classical mass spectrometry–based proteomics has typically been to globally analyze cellular proteins and thereby the processes and functions that depend on them (1). To this end, the field has made tremendous advances over the past decades in proteome and protein coverage as well as in analytical techniques, and proteomics has been used in numerous studies to probe biological processes based on changing protein abundances within a system. Protein abundance changes are, however, limited in the information they can provide about biological regulation, in particular regulation over short time scales. Monitoring changes in protein structure offers a complementary route to probing functional molecular events taking place in a biological system (2).

**Fig. 1:**
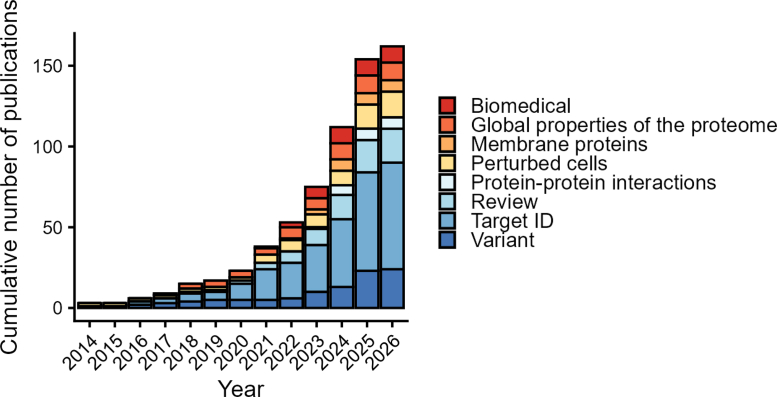
**LiP-MS is being widely applied.** The plot shows the cumulative number of publications using LiP-MS or a variant in every year since the method was described (6) until submission of the revised version of this review. Broad categories of applications or methodological variants are indicated with color. We note that studies where LiP-MS was applied to single purified proteins were not included in this graph.

Protein structure is linked intimately and inextricably with protein function. Within a biological system such as a living cell, proteins undergo frequent conformational switching, meaning that a single protein can adopt various physiologically relevant conformational states (3, 4). These structural changes in proteins take many forms: they can reflect or be induced by changes in molecular interactions, including with DNA, RNA, other proteins, metabolites, metals, or other molecules, including drugs. They can be due to chemical modification (*e.g*., phosphorylation, acetylation), processing by proteases, unfolding, or aggregation. Moreover, certain protein conformations are linked to disease, either as the cause (e.g., conformations induced by mutations or external toxic cues) or as a consequence of altered cellular physiology. Changes in protein structural states therefore encompass information on protein function in both health and disease. Proteomics screens that monitor protein structural in addition to protein abundance changes can therefore much increase functional coverage, rather than merely the number of proteins that can be identified (i.e., proteomic coverage). The field of structural proteomics encompasses several methods aimed at globally investigating the structures of proteins in a system and thus at probing their functional consequences (Box 1) (5). One such method is limited proteolysis coupled to mass spectrometry (LiP-MS), developed in our laboratory over a decade ago (6). LiP-MS is now mature and widely applied (Fig. 1) and thus the subject of this review.

Box 1 **Other structural proteomics techniques**

Distinct from the protease accessibility probed by LiP-MS and its variants, other structural proteomics methods employ chemical labeling or crosslinking mass spectrometry to distinguish protein structural states. In brief, covalent protein painting uses chemical labeling of lysine residues to identify regions with altered labeling patterns upon perturbation (90, 91). This approach pinpoints changes in single lysine residues, and coverage depends on the natural distribution of lysines. Fast photochemical oxidation of proteins employs chemical labeling of all amino acids to screen for structural changes, which offers in principle higher coverage and resolution because each amino acid can be targeted (92). SPROX-MS monitors methionine oxidation over a range of denaturant concentrations, thus providing a peptide-centric readout of protein domain stability (93). Cross-linking coupled to mass spectrometry, in which nearby residues either within or between proteins are covalently linked together via an externally applied crosslinker (94), can also be employed in a comparative fashion to identify structural changes, that is, changing crosslinks, between conditions of interest. Generating the required quantitative cross-linking data for such comparative work, however, remains challenging and the need to identify pairs of linked peptides leads to a dramatic expansion of the search space, complicating statistical control. The specific functional groups (typically primary amines in lysines and at the N terminus) and distances (typically <35 Å) linked in a XL-MS experiment depend on the crosslinker used. Finally, TPP, although not strictly a structural proteomics approach, detects changes in thermal melting curves as a readout for altered protein stability, which includes changes due to altered protein structures (95, 96, 97). TPP has high reproducibility and proteome coverage but, unlike SPROX-MS and LiP-MS, does not provide positional information on the altered protein region or binding site. LiP-MS possesses the attractive combination of high proteome coverage, routinely monitoring structural changes in many thousands of proteins and the ability to pinpoint structural changes with peptide-level resolution. This allows informative mapping of functional sites and formulation of hypotheses about the molecular consequences of a protease accessibility change. Like covalent protein painting and fast photochemical oxidation of protein, however, LiP-MS strictly requires the structurally changing region of a protein to be adequately covered; this can pose a challenge for low-abundance proteins and for poorly accessible protein regions, due for instance to suboptimal trypsin cleavage or to generation of peptides with poor signal response in the mass spectrometer. Further, unlike XL-MS, it does not provide distance restraints for direct use in structural modeling.

**Fig. 2:**
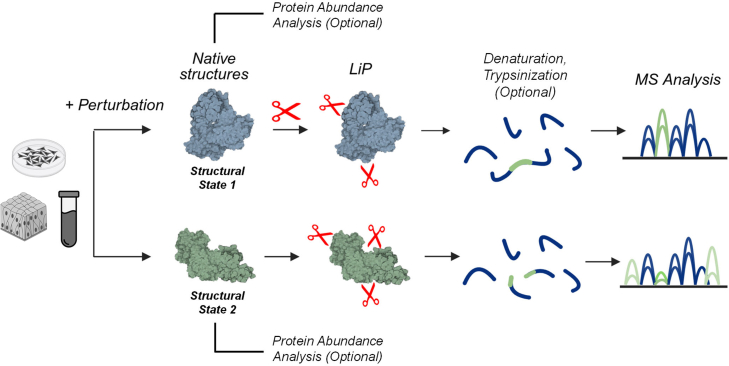
**Global protein structural profiling with LiP-MS.** The schematic illustrates the principle of the LiP-MS approach. In brief, native proteomes extracted by gentle lysis from a sample of interest are briefly exposed to a protease, followed by optional denaturation and trypsinization, and mass spectrometry analysis. Protein structural changes upon an applied perturbation will generate proteolytic susceptibility changes and therefore differences in the protease cleavage pattern relative to an unperturbed control. Optionally, another aliquot of the same proteome is subjected only to trypsin digestion under denaturing conditions to measure and correct for structure-independent protein and peptide abundance changes between treated and untreated samples.

LiP-MS is based on the following principle: if a folded protein is incubated with a protease for a short time, cleavage will occur preferentially in accessible and flexible protein regions (6, 7, 8, 9). Changes in the structural state of the protein, either due to internal cues or external perturbations such as a stressor or a drug, will thus be reflected in changes in the protease cleavage pattern (Fig. 2). In other words, LiP-MS uses changes in the susceptibility to protease cleavage to report on changes in the structural state of a protein.

This limited proteolysis (LiP) principle has been employed since the 1980s to measure aggregation and unfolding of purified proteins, with some studies also using the nomenclature of partial, native-state or pulse proteolysis to describe this method (10, 11, 12, 13). These approaches initially used a simple electrophoretic or chromatographic separation of peptides as a readout, with further variants incorporating peptide sequencing by Edman degradation or mass spectrometry to identify the separated peptides (14, 15, 16). With LiP-MS, which directly couples limited proteolysis and global mass spectrometric analyses, we developed a biochemical and analytical workflow for limited proteolysis on complex samples, that is, on whole proteomes, thus enabling the structural state of thousands of proteins to be probed simultaneously in a context that approximates the complex cellular milieu.

Two precursor approaches of LiP-MS were also based on changes in susceptibility to proteolysis in complex samples, but these again used gel electrophoresis to separate proteins and were therefore not truly proteome-wide: the DARTS (drug affinity responsive target stability) method probed for protected protein bands on a gel upon proteolysis in the presence versus absence of a small molecule or drug and then performed mass spectrometry on the excised bands to identify targets (17). The pulse proteolysis approach analyzed protein stability using LiP under a range of concentrations of the denaturant urea and exploited stability changes upon ligand binding to identify drug and small molecule targets (11). By contrast, we developed LiP-MS to generate peptides that can be directly measured by mass spectrometry on a proteome-wide scale (6).

In a typical LiP-MS experiment (18, 19, 20), condition-dependent protein structural changes are identified by comparing a perturbed sample of interest (e.g., cells or tissues) to an unperturbed control (Fig. 2). Critically, a gentle lysis of the samples is employed to preserve proteins in near-native conditions, before addition of a protease for a short time, typically around 1 to 5 min. Proteinase K (PK) is the most widely used protease in LiP-MS due to its high activity and low sequence-specificity, although other proteases such as thermolysin (21) and subtilisin (22, 23) and even the specific protease trypsin (24) have been used. In principle, also other proteases that have been applied in limited proteolysis experiments on purified proteins, for example, chymotrypsin, elastase, or papain (16) could be used in the LiP-MS setting as well. The LiP step leads to cleavage preferably in flexible, surface-accessible regions. PK activity is then quenched with chaotropes, heating, protease inhibitors, or a combination thereof. Most LiP-MS workflows then use a second digestion step to reduce peptide length, typically with a specific protease like trypsin. Hence, the resulting peptides are either half-tryptic (HT, one end generated by PK and the other by trypsin) or fully tryptic (both ends generated by trypsin). The combined analysis of both these species of peptides has multiple advantages but also introduces some challenges (Box 2).

Box 2 **Analysis of both HT and FT peptides in LiP-MS**

The first version of the LiP-MS method involved a double proteolytic digestion step, first with the nonspecific protease PK under native conditions and then with the specific protease trypsin. This generates both HT and FT peptides, which offers several opportunities. First, analyzing both HT and FT peptides can increase sequence coverage and also makes it more likely that a specific structural change will be detected since many overlapping HT peptides are typically generated. This is particularly useful when an experiment seeks to capture a very localized change, such as upon binding of a small molecule to a protein at a specific binding site. Second, if enough overlapping HT peptides are generated, they can be exploited to pinpoint the structural change at amino acid resolution (98). Third, multiple peptides with changing intensities that map to a given region serve as internal corroboration of the corresponding structural change; therefore, analyzing both FT and HT peptides can strengthen the conclusions of a LiP-MS experiment. In some cases, it is even possible to observe HT and the corresponding FT peptides changing in intensity in different directions, which gives further confidence that a protease accessibility change has occurred in the relevant protein region. However, it should be noted that this pattern cannot be guaranteed, since HT peptides can undergo secondary cleavage, which could alter the direction of peptide intensity changes. For this reason, FT peptides are the more reliable species when the goal is to assess changes in accessibility of a given protein region. In other words, decreased intensity of FT peptides indicates increased protease accessibility (and vice versa) even if the corresponding half tryptic peptide is not detected. The inclusion of HT peptides, however, substantially expands the search space, which intensifies computational demands and increases the risk of spurious identifications. Further, the large number of detected peptides, often with partially overlapping sequences, can complicate aspects of downstream statistical analysis.

Since the perturbation under study may also cause changes in protein abundance, which would impede an accurate comparison of cleavage patterns, samples are typically split into two aliquots and the LiP step is omitted in one of them. Any protein abundance changes introduced by the perturbation can then be identified and corrected. Optionally, peptide (rather than protein) abundance changes that are also not dependent on the exogenously added protease, such as those caused by posttranslational modifications, amino acid alterations, or endogenous cleavage, can also be corrected using these samples (25, 26).

**Fig. 3:**
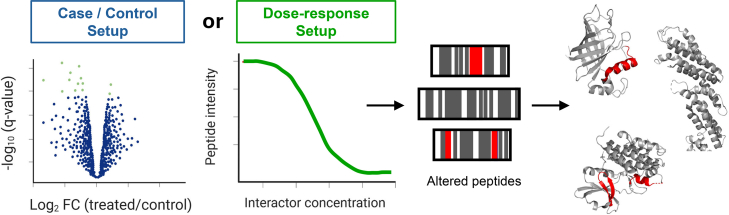
**LiP-MS data analysis to identify structurally altered proteins and interpret structural changes.** Hit peptides are those that differ in intensity between a perturbed and a control condition (case-control setup) or where the peptide intensity shows a dose-dependent response to a dosage series of an added ligand (dose-response setup). Changing peptides may be visualized along protein sequences as linear structural barcodes or mapped onto experimental or predicted 3D protein structures.

This workflow results in a set of peptides that change in intensity due to changes in protease accessibility in the perturbed versus the control condition or that respond in a dose-dependent fashion to an added ligand or stimulus, pointing to changes in structure (Fig. 3). This may be applied in samples ranging from lysates of bacterial, yeast, plant, animal, or human cells to biofluids or tissues from model organisms or human subjects, with the only requirement that the samples can be prepared under near-native conditions. Changing peptides can then be mapped to protein sequences and visualized as linear structural barcodes (2), that is, heat maps that display peptide-level changes along the protein sequence. Changing peptides can also be projected onto experimental or predicted protein 3D structures (Fig. 3). In this way, the peptide-level mass spectrometric readout informs not only about which proteins change upon the perturbation of interest but pinpoints the specific protein regions that show a change in proteolytic susceptibility (i.e., a structural change). LiP-MS has been used with both data-dependent and data-independent acquisition as well as with selected reaction monitoring mass spectrometry and has been applied in labeled and label-free contexts; a recent study compared LiP-MS target identification by DIA versus TMT quantification and compared the use of different analytical tools for DIA search (27). Detailed DIA settings for MS analysis have also been reported (19, 28).

**Fig. 4:**
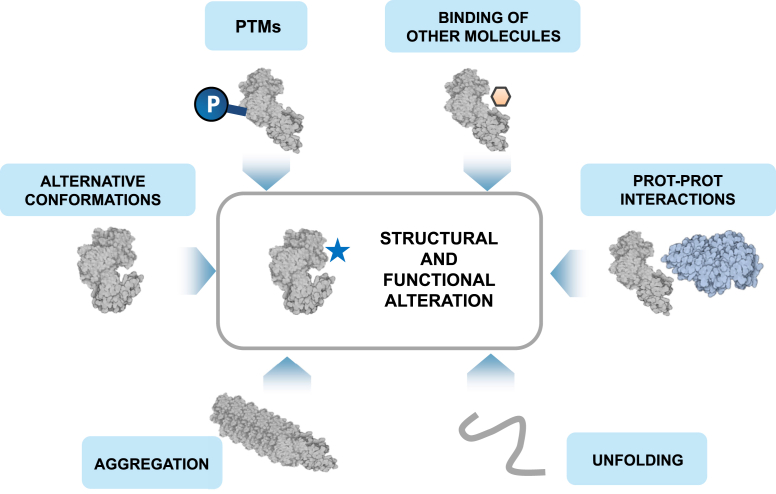
**Protein structural changes integrate many functional molecular events.** The schematic summarizes the classes of molecular events for which changes have been detected by the structural readout of LiP-MS.

Importantly, LiP-MS can capture multiple functional events (Fig. 4): local or global changes in the structural properties of a protein resulting from protein aggregation and unfolding, binding of other proteins, drugs, metabolites and other molecules, PTMs, and conformational changes (2, 6, 29, 30, 31, 32, 33). In samples ranging from unicellular organisms to human biofluids, global profiling of protein structural changes has proven frequently to provide richer information than mere protein abundance changes and captures functional aspects that may be missed by classical proteomics. LiP-MS thus offers detailed insight into cellular regulation at multiple levels, from single protein functional sites up to pathways and the entire proteome. Here we discuss methodological variants of LiP-MS, review its applications, and describe how LiP-MS enable a new type of functional omics analysis that we term 3D proteomics. The many studies applying LiP-MS to single purified proteins are not covered in this review.

## Variants of LiP-MS

**Fig. 5:**
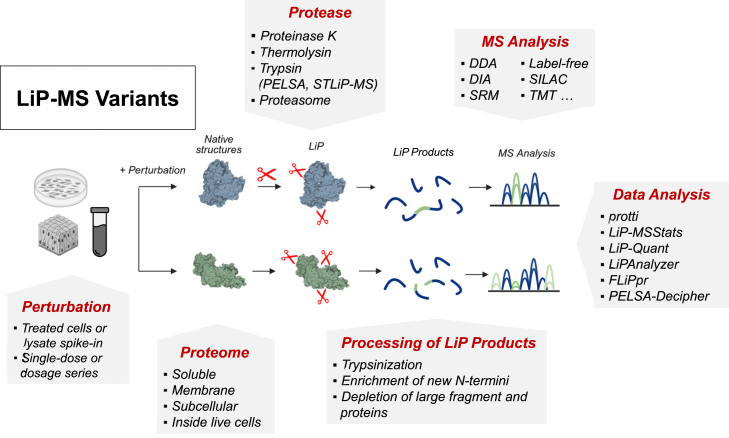
**Methodological aspects that have been varied in different versions of LiP-MS.** These variations have been made both in our own laboratory and those of others. In brief, variations may be in the nature of: the protease, the proteome, the perturbation, and the processing of LiP products. In addition, various types of MS analysis have been used and many software packages developed for LiP-MS.

The first version of the LiP-MS method applied the nonspecific protease PK to a cell or tissue lysate to compare a proteome under perturbation to a control sample in a direct pairwise fashion, with the appropriate number and type of replicates determined by the specific application. This method has been optimized over the years and a recent paper describes the step-by-step procedure (18). We and others have also developed several variants of the method. For the purpose of this discussion, we broadly classify them based on the following: how the perturbation is applied, the protease employed, whether the workflow includes enrichment or depletion of proteolysis products, and the nature of the analyzed proteome/sample (Fig. 5). In this section, we discuss each of these classes of variants. For clarity, we also provide concise definitions of published limited proteolysis-based methods applied to complex samples (Table 1).

**Table 1:** Methods based on limited proteolysis beyond classical LiP-MS

LiPQuant: LiP-MS conducted by applying a dosage series of the ligand or drug of interest rather than a single concentration. Candidate target proteins are identified using various criteria, the most important being goodness-of-fit of the corresponding peptides to the expected dose-response curve. May also be applied to lysates of intact cells that had been treated with a ligand of interest.
In-cell LiP-MS: Variant of LiP-MS where the protease is introduced into intact cells rather than into a lysate. Proteolysis occurs within the cell, followed by cell lysis and processing of peptides as in the classical LiP-MS workflow.
PELSA: Limited proteolysis coupled to mass spectrometry, but using only the specific protease trypsin under native conditions. Named the peptide-centric local stability assay, although proteolysis alone (in the absence of denaturant) cannot strictly probe stability (see text). Like classical LiP-MS, probes changes in susceptibility to proteolysis of native lysates in a pairwise fashion, between two conditions of interest.
STLiP-MS: Swift Trypsin limited proteolysis-mass spectrometry. Again, limited proteolysis is conducted with only trypsin under native conditions, with the trypsin immobilized on a matrix. This permits the use of centrifugation to substantially shorten the limited proteolysis step.
DARTS: Drug affinity responsive target stability. Identifies candidate drug targets by probing for protected protein bands on a gel after proteolysis ± drug, followed by MS analysis of the excised bands.
Pulse proteolysis: Profiles protein stability via analysis of susceptibility to proteolysis in a gradient of denaturant. Stability changes in the presence of a drug are used to identify candidate drug targets.
PepTID: Pulse proteolysis and precipitation for target identification. A variant of pulse proteolysis using acid precipitation to remove undigested proteins after limited proteolysis across the denaturant gradient, followed by direct MS analysis of proteolytic products.
STEPP: Semi-tryptic peptide enrichment strategy for proteolysis procedures. After classical limited proteolysis, fully tryptic peptides are selectively removed from the solution, so that only half tryptic peptides are analyzed by MS.
TALiP-MS: Thermostability-assisted limited proteolysis-coupled mass spectrometry. Lysates ± drug are first subject to thermal proteome profiling (TPP) and the TPP supernatants, which should be enriched in stabilized proteins, are subject to limited proteolysis to identify candidate drug targets.
FLiP-MS: Serial ultrafiltration combined with limited proteolysis-mass spectrometry. Generates a library of markers of protein–protein interactions, including interaction interfaces. The results of any subsequent LiP-MS experiment in the same proteome can then be queried against this library to identify proteins and protein complexes that are likely to change their assembly state.

The principles of the different published limited proteolysis-based methods are briefly described.

### Nature of Perturbation

In LiP-MS experiments where a ligand is added to a system such as a cell lysate to determine its interactors or targets, the ligand may be added at a single concentration as initially described (6) or in a dosage series as in the LiPQuant approach (33). LiPQuant uses machine learning to rank LiP hits according to their likelihood of being true direct interactors of the tested ligand. This approach was first described for ranking putative drug targets in human cell lysates (33) and was later adapted also to ranking protein interactors of a spiked-in protein bait (30). Besides ranking peptides according to how well they follow a dose-response relationship with the ligand of interest, LiPQuant also filters out likely nonspecific peptides based on a “crapome” dataset and weights hits based on the number of peptides per protein that follow a dose-response curve and on the statistical significance of the differential effect of the ligand versus a vehicle control (33). The step-by-step workflow for LiPQuant has been described (19). We note that putative targets/interaction partners identified by either binary comparisons or by LiP-Quant ideally require orthogonal approaches (*e.g*., molecular docking or thermal shift) to validate that hits are direct interactors of the bait of interest rather than proteins that change structure due to indirect effects. But since LiPQuant-like approaches identify direct interactors with higher confidence (33), high-ranked LiPQuant hits are more likely to be validated. Thus, despite being more resource and time intensive, LiPQuant may more rapidly yield robust targets.

**Fig. 6:**
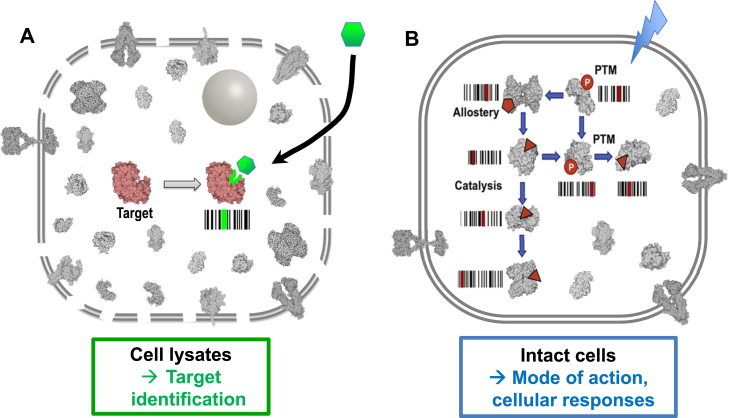
**The design of a ligand spike-in experiment depends on its goals.** A ligand should be spiked into a sample lysate to identify candidate direct interactions, but added to intact cells to profile cellular responses or ligand mode of action.

Further, a ligand of interest may be applied either to intact cells or to a lysate (Fig. 6), depending on the goals of the experiment. For studies that aim to identify direct targets of the ligand, the approach of choice is to add the ligand to a native lysate (32). Especially if the incubation period is short, this results in a higher fraction of hits showing protease accessibility changes specifically due to bait binding and correspondingly fewer changes due to downstream or indirect effects. If the goal on the other hand is the profiling of cellular effects or mechanisms of action of a ligand, it should be added to intact cells, with the caveat that a ligand or chemical perturbation with an intracellular target must be cell permeable; limited proteolysis is then carried out in the native lysate (34).

### Nature of Protease

While PK is the most commonly used protease in LiP-MS studies (6), other proteases have also been used. For instance, thermolysin was applied in a study of protease sensitivity in the *Escherichia coli* proteome under both normal and heat shock conditions, and of the role of the bacterial Hsp70 chaperone DnaK (21). While this work largely used gel electrophoresis of thermolysin digests followed by in-gel trypsin digestion, it also employed the interesting twist of collecting thermolysin-cleaved peptides directly via a filtration step, followed by MS. This latter approach showed that proteins with a c.37 fold (P loop nucleotide triphosphate hydrolases) were enriched in the protease-sensitive set both at normal temperatures and under heat shock. In separate work, the 20S subunit of the proteasome was used as the protease instead of PK in the LiP-MS workflow, to identify cellular substrates of this central endogenous protease under both unstressed and oxidatively stressed conditions. This revealed an enrichment of nucleic acid–binding proteins and of intrinsically disordered regions among the substrates of the 20S proteasome (35).

In an interesting recent development, limited proteolysis using the high-specificity protease trypsin has been extended from its previously established use for LiP on purified proteins (36, 37) to identify structural changes upon ligand binding also in complex cell lysates (24). This approach has been imprecisely named the peptide-centric local stability assay (PELSA), although proteolysis alone cannot be used to infer protein stability, as previously conceptualized (11). Proteolysis alone (i.e., in the absence of a denaturant) under time-limited conditions probes proteolytic susceptibility, which is in turn driven by the accessibility and flexibility of native protein regions as well as by stability. In other words, PELSA is limited proteolysis followed by mass spectrometric analysis but using a specific rather than a low specificity protease. The PELSA workflow has the advantage of simplicity, as it relies on a single protease, and the resulting tryptic peptides benefit from favorable mass spectrometric properties. Also, the resulting mixtures of nonoverlapping tryptic peptides are much less complex than those generated by the double proteolysis step in standard LiP-MS, thus reducing noise. Together, this means that PELSA can perform with high sensitivity for detection of the targets of various ligands (24). A recently described variant, named Swift Trypsin LiP-MS, takes the interesting approach of immobilizing the trypsin on a spin column such that centrifugation can be used to shorten the proteolysis step well beyond what is possible in solution (estimated exposure of <1 s) (38).

However, the use of a specific protease like trypsin for LiP has several disadvantages. First, trypsin must be used at a high enzyme:substrate ratio (1:2 compared to 1:100 for PK in standard LiP-MS), which makes the proteolytic reaction harder to control and thus runs the risk of reducing reproducibility. Second, trypsin cleavage patterns are influenced not only by protein structure but also strongly by protein sequence, biasing the analysis based on the presence and location of arginine and lysine residues. Third, trypsin-based LiP approaches will detect structural alterations of a given region only if the associated tryptic peptide has favorable MS properties (e.g. suitable length and good ionization efficiency). While MS-inaccessible regions are also an issue for classical LiP-MS with dual proteolysis, this problem is expected to occur to a lesser extent. This is because LiP-MS often generates multiple peptides differing by a few residues for a given stretch of protein sequence, which increases the probability of at least one peptide with favorable MS properties.

### Processing of Proteolysis Products

It has been shown that enrichment of HT peptides can increase protein coverage and structural information content of LiP-MS screens (39). In this approach, amino groups of peptides that have undergone LiP are first protected by reaction with isobaric mass tags, followed by digestion with trypsin. Tryptic peptides (*i.e.*, with unprotected N-termini) are then removed from the solution, leaving only HT peptides for MS analysis. Such an approach, termed STEPP, identified 5-fold more breast cancer–related HT peptides than regular LiP-MS in a comparison of human cell lines and has also been combined with pulse proteolysis (39, 40). Separately, the PELSA approach uses a depletion step, in which undigested proteins or large fragments are removed by filtration after proteolysis (24), and a variant of pulse proteolysis called PePTID uses acid precipitation to remove undigested proteins after LiP across a urea gradient, allowing direct MS analysis of proteolytic products (41). In a recent study, intact protein mass spectrometry was used to directly quantify large limited-proteolysis products of KRas, while top-down analysis enabled their identification (42). This approach supported both localization of the cleavage site and quantification of cleavage extent; however, its scalability to complex proteome extracts in an LiP-MS setup remains to be determined.

### Nature of Sample

The standard LiP-MS approach is applied after gentle cell lysis. However, depending on the subset of the proteome that is of interest, these lysates may be cleared of insoluble material by centrifugation, which will remove many membranes and membrane proteins, or may be left uncleared (33). For experiments focusing on membrane proteins, crude preparations of membranes have been used (43). Most recently, our laboratory has developed in-cell LiP-MS, using electroporation of active PK into human cells and thus enabling the study of protein structural changes in a truly native environment, that is, within intact, living cells rather than in a cell lysate (44). This approach captures stable interactions like the rapamycin-FKBP1A drug-target interaction, as does standard LiP-MS, but also enables the study of labile assemblies like biomolecular condensates that may dissociate partially during cell lysis. We used in-cell LiP-MS to investigate the formation of stress granules upon arsenite treatment of human cells, providing an unprecedented time-resolved picture of protein structural alteration for hundreds of stress granule proteins simultaneously, with peptide-level resolution.

## Tools for LiP-MS Data Analysis

The LiP-MS data analysis workflow starts with raw mass spectrometry files, which are processed using established software such as Spectronaut or the open-source FragPipe suite (45), to identify peptides and precisely quantify their intensities. Peptide intensities from LiP-MS applied to perturbed cells or tissues reflect a combination of protein structural and abundance contributions, requiring dedicated analysis methods to disentangle these effects and perform statistical analysis at the peptide level. Therefore, several specialized software packages have been developed to address the statistical challenges of LiP-MS data. MSstatsLiP (18), an R package, provides a robust statistical framework designed to detect structurally altered peptides between conditions. Crucially, it distinguishes structural changes from abundance changes by correcting for protein abundance variations when a corresponding tryptic control dataset is available. The protti R package implements the same abundance correction approach and additionally includes specialized modeling for dose-response LiP data. Protti also offers extensive quality control features and advanced visualization tools, such as structural barcodes and the mapping of altered peptides directly onto 3D protein structures (46), making it ideally suited to support a broad range of users, including those who are new to LiP-MS data. Addressing the challenge of missing data in proteomics, FliPPR (integrated within FragPipe) offers missing data imputation strategies tailored specifically for LiP-MS and aggregates data at cleavage sites using rigorous site-level statistics (47). Similarly, the open-source library ProteoMeter integrates LiP-MS data with other structural proteomics modalities, such as PTM profiling and cross-linking mass spectrometry, by quantifying changes specifically at cleavage sites (48). Finally, LiPAnalyzeR applies regression models to identify structural changes while removing unwanted variation arising from confounding factors like alternative splicing or PTMs (26). This R package further provides a statistical framework optimized for large-scale datasets, including human clinical cohorts (25).

## Applications of LiP-MS

### Protein Conformational Changes in Perturbed Systems

Given its ability to monitor structural dynamics across entire proteomes, LiP-MS has been harnessed in numerous studies in our own and various other laboratories to profile systems under a perturbation of interest. In early work, we performed the first global structural analysis of a cellular proteome in yeast grown on glucose versus ethanol. We observed that regulation of specific branches of metabolic pathways was captured exclusively by the structural readout, as the corresponding enzymes did not undergo changes in abundance and therefore escaped detection by classical protein expression analyses (6). This gave early support to the notion that a structural readout has the potential to dramatically expand the reach of functional proteomic screens. We systematically demonstrated this in yeast responding to brief osmotic or heat shock, where only few protein abundance changes were seen at early time points after perturbation but structural alterations were already widespread (2). Similar patterns were reported by others, for instance, in a pulse proteolysis study of *E. coli* exposed to copper with and without ionophores (49). Importantly, changing LiP peptides upon yeast osmotic shock mapped to known altered functional sites of the proteins involved, such as regulated phosphorylation sites, catalytic sites, and allosteric sites within the same pathway (2), illustrating that the method can detect protease accessibility changes due to multiple types of structural rearrangements simultaneously and pinpoint altered locations on protein structures.

Protein structural dynamics have been profiled by LiP-MS in numerous other perturbed systems. For instance, LiP-MS has identified three transcription factors that sense changes in L-arginine levels in activated T cells and promote their survival (50). In lipopolysaccharide-activated macrophages, LiP-MS identified conformational changes in proteins associated with protein folding, like HSP60, which could be confirmed by thermal shift (51). In lung-derived cell lines and primary cells, LiP-MS revealed that alpha-coronaviruses use a multifaceted strategy targeting several RNA-processing pathways to co-opt host cell function (52). In budding yeast, it identified aging-associated changes in multiple cellular processes, including translation, protein folding, and amino acid metabolism, and led to the discovery that the enzyme glutamate synthase polymerizes into supramolecular self-assemblies during aging (53).

In work on *E. coli* central carbon metabolism, we probed how LiP data could be integrated with other information to functionally interpret protease accessibility changes. Specifically, we applied LiP-MS to bacteria grown in medium with each of eight different carbon sources and then correlated abundance-corrected LiP-MS data with metabolic flux data from cells under the same conditions (2). This identified LiP peptides from a few glycolytic enzymes that behaved as quantitative predictors of flux, likely because changing substrate occupancy in different growth conditions changed the susceptibility of the relevant enzyme to protease cleavage. Further, by correlating LiP peptide intensities with measured metabolite concentrations under these growth conditions and integrating prior knowledge about protein–metabolite interactions, we could predict allosteric regulation of several enzymes in central carbon metabolism during nutrient adaptation and enabled identification of a novel regulatory mechanism in this pathway.

### Disease Mechanisms and Biomarker Discovery

Protein structural changes occur not only under external perturbation but should also accompany (and in some cases cause) pathophysiological changes in disease. Studying such structural alterations with a global molecular readout is therefore a promising approach to characterize disease mechanisms. LiP-MS has been used, for example, to probe the cellular effects of disease-associated calreticulin mutations in patient samples and in cell lines derived from individuals with myeloproliferative neoplasms (54). This showed that homozygous (but not heterozygous) calreticulin mutations perturb the glycoproteome, suggesting that the loss of the functional chaperone caused glycoprotein maturation defects. As another example, LiP-MS was used alongside thermal proteome-profiling (TPP) and stability of proteins from rates of oxidation (SPROX)-MS to identify proteins that changed structure and thermal stability in oxaliplatin-sensitive versus oxaliplatin-resistant cancer cell lines and patient-derived xenografts (55). In a LiP-MS study of mouse CSF, aging-associated structural changes were identified with high confidence in six protein hits with associations to human neurodegeneration (56).

Importantly, global structural analyses by LiP-MS have enabled new directions in biomarker discovery. Most proteomics-based biomarker screens aim to identify disease-associated changes in protein abundance or chemical modifications, but are blind to structure-associated alterations. Recently, we illustrated how LiP-MS enables the novel concept of structural biomarkers of disease (25). Using LiP-MS combined with statistical modeling to correct for covariates, we identified 76 proteins with protease accessibility changes in cerebrospinal fluid of a cohort of Parkinson’s disease (PD) patients relative to healthy subjects. Notably, 16 of these proteins were also altered in the brain samples of PD patients and could therefore represent changes directly relevant for pathophysiology. While many of these hit proteins belonged to classes known to be involved in PD (*e.g.*, synaptic proteins, neurotransmitter receptors), the structural changes identified were novel, so that this study single-handedly increased the number of structurally altered proteins that may be associated with PD, the second-most prevalent neurodegenerative disease, by more than an order of magnitude. Further, it demonstrated again that protein structural information can be much more informative about changes in a system than abundance information and also provided as a side product the first detailed analysis of the extent of protein structural variability within the proteome of a healthy human cohort.

This work demonstrated the new concept of structural biomarkers in PD, where the lack of substantial protein abundance changes in patient biofluids has hampered biomarker discovery. It is applicable, however, to both biomarker discovery and mechanistic studies in other diseases as well. Indeed, LiP-MS has since been used to report structurally altered regions in 12 CSF proteins (57) and 23 serum proteins (58) in individuals with Alzheimer’s disease or mild cognitive impairment, relative to healthy subjects. In more recent work, hippocampal lysates from mice with Alzheimer’s-associated mutations were investigated with LiP-MS, TPP, and the related SPROX to detect many hundreds of structurally altered proteins in mutant mice relative to WT (59). Two hundred to five hundred proteins showed changes per technique but overlap between techniques was low, as expected given the different mechanisms of their readouts. Interestingly, 3 of the 25 high-confidence changes (detected by at least two techniques and at two time points) overlapped with hits of a previous LiP-MS study in human (57). Investigating a different biofluid (i.e., saliva), a LiP-MS study of oral squamous cell carcinoma found structural changes in 36 proteins that were correlated with clinical features (60). Taken together, global structural screens by LiP-MS offer a novel molecular readout for human pathological states and thus a promising new avenue for biomarker discovery and for mechanistic studies of disease.

### Global Properties of the Proteome

The ability of LiP-MS to probe structural changes in complex cellular mixtures has been harnessed to identify general properties of the proteome. Broadly speaking, if one identifies the subset of a proteome that structurally responds to a given perturbation (e.g., temperature, dephosphorylation) or that possesses a particular quality (e.g, refoldability), then the sequence, structural, or biophysical properties of the proteins that comprise that subset can be analyzed to reveal determinants of the feature being tested or can be used to train machine learning models to predict that feature.

For example, we used LiP-MS under a temperature gradient to identify evolutionarily conserved determinants of protein thermostability (31). The resulting dataset comprised stability measurements for >8000 proteins in four species and was used by others to train a predictor of protein thermostability (61). In separate work, re-analysis of a LiP-MS dataset of yeast subjected to heat shock (2) used a machine learning approach to identify features of aggregation-prone regions in the 96 proteins that showed significant structural changes under these conditions (62). This work revealed that aggregation-prone regions are enriched in aliphatic residues and depleted in positively charged amino acids and that the length of these regions influences a protein’s propensity to aggregate. The ability of LiP-MS to probe thermal stability has also enabled the discovery that osmolytes of several classes stabilize hundreds of proteins in the *E. coli* and the human proteomes (29) and has helped dissect the mechanisms of action of osmolytes. Specifically, osmolytes were found to act via a preferential exclusion mechanism, that is, by rendering the unfolded state energetically unfavorable via interacting with the protein backbone. LiP-MS has also been used to study response of the yeast proteome to desiccation and resolubilization and thus help characterize the inherently dessication-tolerant subset of the proteome (63). Dessication-tolerant proteins (i.e., those that tend to resolubilize) are likely to retain their structure after this manipulation. This set of proteins was found to be enriched in negative surface charge and was more likely to include proteins that produce small molecules, in keeping with a functional need for such proteins upon dessication-rehydration.

In a recent interesting application, a device was developed to implement LiP-MS at the high pressures extant in deeper layers of the ocean and found that, contrary to previous work that monitored global unfolding, a substantial (nearly 40%) of the *Thermus thermophilus* proteome showed local structural changes at these pressures (64). This was influenced by protein packing density, isoelectric point, and co-factor binding and thus provides a first hint of determinants of pressure sensitivity in proteins within their native context. Further, in combination with SILAC, LiP-MS has identified protease accessibility changes in more than 200 proteins upon global dephosphorylation in a breast cancer cell line (40, 65); this is of particular interest since the functional consequences of (de)phosphorylation are relatively understudied and probing the dephosphorylation-sensitive proteome may provide insights into this question.

Finally, a series of elegant studies have used LiP-MS on refolded samples. By analyzing re-folded proteins in *E. coli* lysates upon dilution or dialysis of an added denaturant, the effect of the chaperones GroEL or DnaK on protein folding could be studied. Interestingly, one-third of the proteome was found to be nonrefoldable, even in the presence of these chaperones, and the properties of these proteins suggest that they may be obligate cotranslational folders (66). These data were subsequently re-analyzed in parallel with a molecular dynamics simulation study to show that synonymous mutations in the *E. coli* ribonuclease can affect dimerization via formation of an entangled state, which depends on translation rate (67). Indeed, such entangled states, which bypass the proteostasis/quality control machinery but can show reduced protein function, have been shown by molecular dynamics simulations to be highly prevalent (about a third of the *E coli* proteome based on simulation of a subset of the proteome); parallel in-lysate LiP-MS experiments could validate these simulated states for glycerol-3-phosphate dehydrogenase as a case study (68).

Thus, LiP-MS can shed light on general physical properties of the proteome and identify protein regions associated with a property or behavior of interest.

### Identification of Drug and Metabolite Targets

Binding of a small molecule to a protein can alter protease accessibility of the binding site via steric hindrance and/or can result in allosteric changes in the protein structure that alter accessibility also at some other protein region. Either of these events would be expected to change the cleavage pattern of a ligand-bound versus unbound protein. This was first exploited for target identification in the DARTS and pulse proteolysis methods, initially using an electrophoretic readout (11, 17).

By coupling LiP to global MS analyses, target identification via limited proteolysis was rendered a proteome-wide discovery technique. In the first systematic study of this type, we profiled by LiP-MS proteins changing protease accessibility across the proteome after individual addition of 20 metabolites to a native *E. coli* lysate (32). This approach captured both known and novel protein–metabolite interactions and, exploiting the peptide-level resolution of LiP-MS, pinpointed their binding sites. The study also demonstrated that in-lysate protein-metabolite binding affinities could be estimated by applying LiP-MS to samples treated with a range of metabolite concentrations. We developed this dose-response concept further in developing the LiP-Quant method to study drugs targeting kinases, phosphatases, and membrane proteins in human cells (33); in separate work, LiP-Quant was also shown to correctly identify the target of a known CDK inhibitor and map its binding site (69). Further, LiP-MS has been used to profile tomato root lysates for putative ATP-interacting proteins (70), identify interactors of the mushroom toxin α-amanitin in human hepatocytes (71), and investigate how methylerythritol cyclodiphosphate, a metabolite in bacteria and plants, affects *E. coli* biofilm formation (72). In this work, comparison of metabolite-treated and untreated lysates identified the protein H-NS as its direct interactor, with confirmation using protein thermal shift assays. Methylerythritol cyclodiphosphate was further shown to modulate H-NS activity, which affects fimbriae production and thereby *E. coli* biofilms. Most recently, we have shown that LiP-MS can even identify proteolytic susceptibility changes due to the binding of metal ions (ionic radius of 0.6–0.8 Å) (73).

LiP-MS has found perhaps its widest current application in the identification of drug targets in native lysates, predominantly for compounds from natural sources and with an unknown molecular mechanism. It has been used, for instance, to identify Dlat as a direct target of hyperforin, a plant metabolite with anti-obesity properties (74), and hexokinase 2 as a direct interactor of traumatic acid, an anti-oxidant plant metabolite that prevents lipid accumulation in human adipocytes (75). LiP-MS has further identified protein phosphatase 2A as a direct target of lomitapide in human colorectal cancer cells (76), HSP90AA1 as the target of flavonoids used for treating Hashimoto’s thyroiditis (77), human angiotensin-converting enzyme 2 as a target of benzoylaconitine, a plant compound used for the treatment of cardiovascular diseases (78), and in combination with MRM was used to help validate annexin A6 as a DARTS-identified target of a novel thiadiazolopyrimidone (79). Later iterations of pulse proteolysis, in which LiP is applied across a urea gradient, have also been coupled directly with mass spectrometry for global analyses of protein stability and used for target identification, for instance to identify NAD + binding proteins in the *E. coli* or ATP-binding proteins in the *Mycobacterium smegmatis* proteome (41, 80).

In a similar fashion, LiP-MS has been used to profile protein–lipid interactions, with the difference that lipids were added to the lysate in an immobilized context, such as on beads. Putative interactors of phosphatidylinositol 4,5-bisphosphate were identified by screening for proteins that underwent structural changes upon addition of phosphatidylinositol 4,5-bisphosphate–conjugated agarose beads to a nuclear lysate of HeLa cells, relative to blank control beads (81).

Further, LiP-MS has been used to define putative binders to inorganic materials: a time-course of LiP with trypsin described layers of the protein corona formed on magnetic nanoparticles from a wheat germ extract (82), and classical LiP-MS was used to identify candidate interactors of silica nanoparticles in rice leaf extract (83) and of microplastics in umbilical cord blood (84), in both cases upon particle spike-in to the respective lysate.

Given the complementarity of TPP and LiP-MS for target identification (i.e., identifying changes in thermal stability vs in proteolytic accessibility), each technique may serve as an orthogonal validation for the other, with LiP-MS additionally providing information about the potential interaction site. For instance, both techniques have been applied to characterize MIPS2673, a selective inhibitor of the Plasmodium M1 alanyl metalloaminopeptidase (85): both techniques identified the expected target and potential off-targets, with several of the latter being metalloproteins as expected. The changing LiP-MS peptides further provided a good approximation of the inhibitor binding site on the enzyme, mapping within 5 Å of that determined by X-ray crystallography. In another study, thermal shift assays identified interactions between proteins and rocaglates, a family of molecules clamping specific protein–RNA interactions, and LiP-MS successfully validated the identified target (86). Finally, the two techniques have been combined in an approach termed thermostability-assisted limited proteolysis-coupled mass spectrometry, to increase sensitivity and efficiency in drug-target identification (87). Here, cell lysates treated or not with a drug of interest are processed by TPP (i. e., centrifugation of samples subject to a temperature gradient) to identify proteins enriched in the supernatant and therefore likely stabilized, in the presence of the drug. These supernatants are then subject to limited proteolysis. This approach was tested on the drugs cyclosporin A, geldanamycin, and staurosporine and increased the number of known target peptides over a standard LiP-MS experiment.

In sum, LiP-MS may be used both for discovery and for validation of direct interactors of drugs, metabolites, and other small molecules, with orthogonal validation typically required in the discovery context.

### Identification of Protein–Protein Interactions

The use of LiP-MS to identify molecular interactions is not limited to protein-small molecule binding events but has been applied to identify protein–protein interactions and protein interaction interfaces as well. Broadly speaking, LiP-MS–based methods to study PPIs have followed one of two strategies. The first is based on spike-in of the bait protein of interest, for which we demonstrated proof of principle using conformation-specific antibodies against the respiratory syncytial virus F glycoprotein. This study showed that LiP-MS on native HEK293T lysates treated with a dosage series of the antibody motavizumab could identify altered peptides on the expected target that moreover mapped at or near the known epitope (30). Such an approach also lends itself well to studies of conformation-specific PPIs, as we showed by spiking monomeric or fibrillar forms of the PD hallmark protein alpha-synuclein into cell lysates to identify such putative differential interactors. Using a similar strategy, LiP-MS has been used to show that the ASC protein, involved in the system human disease inflammation-induced amyloidosis, interacts with serum amyloid A both *in vitro* and in cell lysates and mapped the interaction interface to the ASC pyrin domain (88). Together with the findings discussed in the previous section, LiP-MS has thus proven able to capture interactions of proteins with binders over a wide range of sizes, extending from relatively large nanoparticles to individual metals.

The second strategy using LiP-MS to study PPIs is based on the concept of identifying peptide markers of protein–protein interactions; these can then be used to monitor protein complex dynamics in any subsequent LiP-MS experiment (34). Dynamics of protein complexes are notoriously difficult to study, since classical MS-based methods to profile PPIs, such as SEC-MS and AP-MS, are laborious and can be prohibitively difficult to implement at scale, for example, under multiple conditions. As an alternative and much more scalable approach, we used serial ultrafiltration to separate large protein complexes from their monomeric subunits and then comparatively analyzed the fractions by LiP-MS. Comparing cleavage patterns for the same protein in fractions containing protein complexes with fractions containing largely monomeric forms yielded a library of peptides that differ between monomeric and complex-bound forms of proteins and thus serve as marker peptides for changes in PPIs. This library was enriched in protein–protein interfaces, since protein regions at interfaces are more likely to be protected from proteolytic cleavage. We termed this approach FLiP-MS; importantly, this FLiP library can then be intersected with a separate LiP experiment to identify protein complexes that changed their assembly state under these conditions. In our work, we used FLiP-MS to probe structural changes under DNA replication stress in yeast, in both WT as well as in a mutant lacking acetyltransferase activity of the SAGA regulatory complex, which is implicated in the response to stress (34). Coupled with network propagation approaches, this pinpointed a set of protein complexes that are likely altered under these conditions and revealed a novel role of the SAGA protein complex in the response to DNA replication stress. This strategy thus offers a powerful and new way to generate hypotheses on PPI dynamics at high throughput.

Finally, the ability of LiP-MS to identify protein–protein interaction interfaces has been exploited in hybrid structural approaches to help define contact sites between interacting proteins. LiP-MS is complementary to techniques such as cryo-EM since it can be applied under native conditions and is well suited to study the flexible domains that are difficult to resolve in reconstruction-based methods. LiP-MS was applied to suspensions of rod outer segment membranes incubated with increasing concentrations of calmodulin, confirming under native conditions the interaction of calmodulin with the CNGA and CNGB subunits of cyclic nucleotide–gated channels that had been observed in cryoEM *in vitro* (89). In another study, LiP-MS identified contact sites between the membrane adenylyl cyclase AC8 and its interactors that occur in the flexible regions of AC8 and were therefore not resolved in the cryo-EM structure (43). This allowed the inference that the interactors calmodulin, Gαs, and Gβγ cause different conformational changes in AC8.

### Application to Membrane Proteins

As for all MS-based methods, the study of membrane proteins presents a challenge for LiP-MS, since these proteins are often lost during sample preparation and/or are at relatively low abundance. Nevertheless, with appropriate steps, protease accessibility changes in this interesting class of proteins can be captured by LiP-MS. For instance, we have shown that when LiPQuant is conducted on human cell lysates that are not centrifuged to remove insoluble material, the number of detected membrane-associated proteins can be doubled (33). This study thus correctly identified the known target of the drug proscillaridin A as the sodium/potassium-transporting plasma membrane pump ATP1A1. In an alternative strategy, where the goal was to specifically investigate interactions with the membrane-integral adenylyl cyclase type 8 (AC8), this could be achieved by probing crude membrane preparations of human cells expressing this protein (30). Such preparations have also been used in LiP-MS–based mapping of structural changes in flexible regions of AC8 and of cyclic nucleotide–gated channels upon calmodulin and/or G protein binding (43, 89).

## 3D Proteomics: From LiP-MS Data to Biological Meaning

**Fig. 7:**
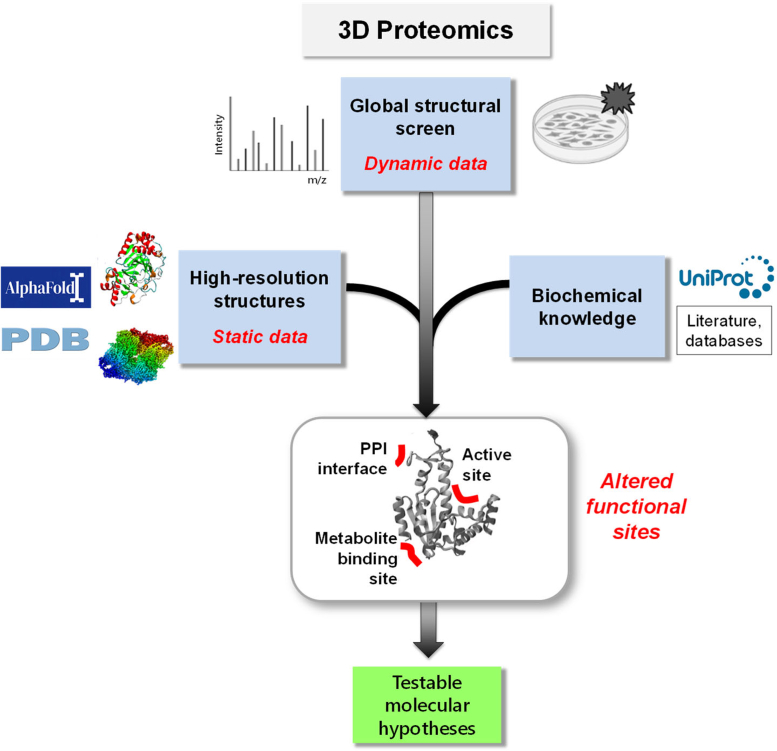
**3D Proteomics.** Integrating proteome-wide structural dynamics data (such as LiP-MS data) with high-resolution 3D protein structures and prior biochemical knowledge constitutes a new type of functional omics screen. 3D proteomics generates a wealth of molecular hypotheses about structural and functional changes in individual proteins with peptide-level resolution and much enriches the systems-wide functional information that can be obtained in proteomics screens.

Global structural analyses enabled by LiP-MS constitute a new molecular readout for protein function that can be applied proteome-wide within native or near-native biological systems. On the basis of this readout, we propose a new omics workflow for functional proteomics screens, which we term 3D proteomics (Fig. 7). In brief, 3D proteomics starts with a global screen for altered structures under a perturbation of interest, followed by mapping of the data to static 3D experimental or predicted protein structures and incorporation of prior biochemical knowledge for interpreting the nature of the detected structural changes. This concept is not restricted to LiP-MS data but would apply to any experimental approach with similar technical capabilities.

There are broadly two ways in which such a 3D proteomics workflow can be used to gain biological insight. First, such data powerfully complement the classical, abundance-based proteomics readout, from which it is often challenging to generate hypotheses about affected processes. A readout of protein structural dynamics integrates information from the myriad functional molecular events that affect protein structure in a single analysis. Thus, in combination with protein expression analysis and prior knowledge, for example, functional enrichment analyses, 3D proteomics data can be used to capture changing pathways and processes with substantially increased sensitivity than classical proteomics alone (2).

Second, 3D proteomics data can be used to pinpoint changing protein regions under a condition of interest with at least peptide-level resolution. By mapping changing LiP peptides to static experimental or, increasingly, predicted protein structures, one may assess the proximity of regions with altered protease accessibility to known functional sites. A changing LiP peptide mapping at an enzyme active site, for example, suggests altered substrate occupancy and thus altered enzyme activity in the tested condition (2). Similarly, altered accessibility of a protein–protein interaction interface suggests assembly alterations of the corresponding protein complex (34). Thus, 3D proteomics data are a rich source of molecular hypotheses about functional events that may occur upon a specific perturbation or in a specific state, such as a disease state, as demonstrated by the range of novel discoveries they already enabled.

In sum, and as our review of the literature illustrates, 3D proteomics data with LiP-MS can provide information on numerous biological problems, ranging from disease-associated structural changes to systems-wide changes during complex cellular perturbations to very specific drug–target interactions. The LiP-MS protocol is a simple addition to existing pipelines and can thus be easily added to functional proteomics screens based on protein abundances to greatly increase the coverage of either physiological or pathological structural alterations. In the future, we expect that the concept of structural markers that we have illustrated for PPIs will be extended to additional molecular events, such as protein-metabolite binding or enzyme activity.

## Conflicts of interest

P. P. is an inventor on patents that cover variants of the LiP-MS approach. All other authors declare that they have no conflicts of interest with the contents of this article.
